# Overcoming Therapeutic Resistance of Bone Sarcomas: Overview of the Molecular Mechanisms and Therapeutic Targets for Bone Sarcoma Stem Cells

**DOI:** 10.1155/2016/2603092

**Published:** 2016-12-27

**Authors:** Tomohiro Fujiwara, Toshifumi Ozaki

**Affiliations:** ^1^Department of Orthopedic Surgery, Okayama University Graduate School of Medicine, Dentistry, and Pharmaceutical Sciences, Okayama 7008558, Japan; ^2^Department of Intelligent Orthopaedic System, Okayama University Graduate School of Medicine, Dentistry, and Pharmaceutical Sciences, Okayama 7008558, Japan

## Abstract

Bone sarcomas are heterogeneous malignant tumors that exhibit clinical, histological, and molecular heterogeneity. Recent progress in their multimodal treatment has gradually improved patient prognosis; however, drug resistance and distant metastasis remain unresolved clinical problems. Recent investigations have suggested the existence of cancer stem-like cells (CSCs) in bone sarcomas, which represent a subpopulation of tumor cells with high tumor-forming ability. The hallmarks of CSCs include tumor- and metastasis-forming potential and drug resistance, which are responsible for poor prognoses of bone sarcoma patients. Therefore, elucidation of the molecular mechanisms of CSCs and identification of therapeutic targets could contribute to novel treatment strategies for bone sarcomas and improve patient prognosis. This paper provides an overview of the accumulating knowledge on bone sarcoma stem cells and preclinical analyses to overcome their lethal phenotypes, in addition to a discussion of their potential for novel therapeutics for bone sarcomas.

## 1. Introduction

Bone sarcomas are a heterogeneous group of malignant bone tumors characterized by various degrees of mesenchymal differentiation. Since their origin has not been identified, bone sarcoma classification is based on morphological findings, such as cell type, architecture, and matrix production. The World Health Organization (WHO) system is generally accepted as the basis for bone sarcoma classification [1]. Bone sarcomas constitute 0.2% of all malignancies in adults and approximately 5% of childhood malignancies, as determined by the Surveillance, Epidemiology, and End Results (SEER) study. Cancer registry data with histological stratification indicate that osteosarcoma is the most common primary bone sarcoma, constituting approximately 35%, followed by chondrosarcoma with 25%, and Ewing sarcoma with 16% [2].

Osteosarcoma is the most common primary malignant tumor of bone with a peak incidence in adolescents and young adults. With combined treatment (neoadjuvant chemotherapy, surgery, and adjuvant chemotherapy), the 5-year survival rate for patients with no metastatic disease at diagnosis is 60%–80% [3–5]. However, for poor responders to chemotherapy and patients with metastatic disease, outcomes are far worse at <50% and <30% survival, respectively [3, 6]. The survival rate has hardly improved for 20 years despite multiple clinical trials. Likewise, the current chemotherapy protocols used to treat Ewing sarcoma, the second most common sarcoma of bone in children and young adults, include various combinations of the following six drugs: doxorubicin, cyclophosphamide, vincristine, actinomycin-D, ifosfamide, and etoposide. Biologically, Ewing sarcoma is characterized by recurrent balanced translocations involving the EWSR1 gene and a member of the ETS family of transcription factors, most commonly FLI-1 [7]. Although multidisciplinary care incorporating advances in diagnosis, surgery, chemotherapy, and radiation has substantially improved the survival rate of patients with localized Ewing sarcoma to nearly 70% [8], survival in a metastatic or recurrent disease setting remains extremely low at <20%. Chondrosarcoma, a malignant group of cartilaginous matrix-producing neoplasms typically occurring in the fifth to seventh decades of life, is generally resistant to chemotherapy and radiotherapy, while Ewing sarcoma is relatively sensitive [1]. Its prognosis depends largely on the histological grade and treatment is mostly limited to surgical resection [9].

The clinical outcomes of these bone sarcomas have plateaued for the last 10 years. Considering the characteristics and heterogeneity of bone sarcomas, it is possible that a subset of tumor cells might resist various stresses and produce recurrence or metastasis, which corresponds to the hallmarks of cancer stem-like cells (CSCs). Indeed, there are no fewer bone sarcoma cases involving metastases long after initial treatments [10]. Although targeted therapy for bone sarcoma stem cells has not been available, several preclinical trials have been reported, which might improve patient prognosis. This paper provides an overview of the accumulating knowledge on bone sarcoma stem cells and preclinical analyses to overcome their lethal phenotypes.

## 2. Cancer Stem Cell Hypothesis in Bone Sarcomas

**Figure 1:**
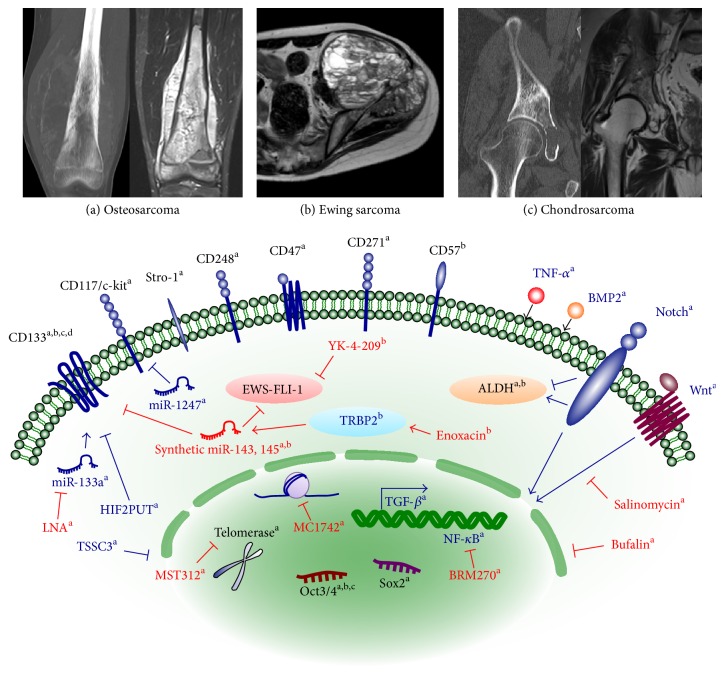
Overview of bone sarcoma stem cells. Although there is no consensus on the definitive marker, a wide range of CSC markers (black) and the molecular mechanisms underlying CSC phenotypes (blue) have been documented for each sarcoma. Several anti-CSC compounds (red) have been preclinically tried to inhibit CSC phenotypes. a, osteosarcoma; b, Ewing sarcoma; c, chondrosarcoma.

The cancer stem cell hypothesis is based on the observation that not all cells in tumors are equal [11]. It proposes that there is a small subpopulation of cells within a heterogeneous tumor that are responsible for forming the bulk of the tumor [12, 13]. These cells are similar to normal stem cells and may arise from the transformation of stem cells or the dedifferentiation of nonstem cells [14]. The common consensus is that they are able to self-renew and differentiate into all of the cells within a tumor [12]. The first evidence of the existence of CSCs was reported in hematological malignancies [11], with these cells being characterized as the CD34^+^CD38^−^ fraction [15]. CSCs have now been isolated from various human solid tumors, including bone sarcomas [13]. The first demonstration of the existence of bone sarcoma stem cells was achieved by Gibbs et al. in 2005, who showed that osteosarcoma and chondrosarcoma cells include a subpopulation of cells that are capable of growing in spheres and have the properties of self-renewability and multipotency [16]. Thereafter, several CSC markers that are common to other malignant diseases as well as unique to bone sarcomas have been identified (Figure 1). Recent investigation has focused on the molecular mechanisms underlying bone sarcoma stem cells and therapeutic testing using preclinical models.

## 3. Characterization of Bone Sarcoma Stem Cells

Since the first identification of osteosarcoma stem cells by Gibbs et al. in 2015, various reports on osteosarcoma and Ewing sarcoma stem cell have been reported while being limited for chondrosarcoma. Most of these reports have documented the conventional CSC markers such as CD133, ALDH, and side population. Notably, the mesenchymal stem cell markers such as CD117/Stro-1 and CD273 have been included, which are the unique characteristic of bone sarcoma stem cells (Figure 1, Table 1). However, there is no clear consensus on the definite marker that characterizes bone sarcoma stem cells, and discussions of several markers, such as side population, have caused controversies in the literature.

### 3.1. Sarcosphere (Osteosarcoma, Ewing Sarcoma, and Chondrosarcoma)

Gibbs et al. first reported that several osteosarcoma cell lines established from biopsy specimens and MG63 OS cell line formed spheres at a frequency of 0.1%–1% in an anchorage-independent environment, which could form secondary spheres after the dissociation of single cells [16]. Wang et al. also identified sphere formation from OS99-1, MG63, HuO9, and SaOS2 [17], and Wilson et al. found similarities between human and canine osteosarcoma cell lines in terms of forming spheres [18]. Fujii et al. further identified that MG63 spheres showed resistance to doxorubicin and cisplatin and increased expression of the DNA repair enzyme genes MLH1 and MSH2, indicating that a DNA repair inhibitor had the potential to enhance the efficacy of chemotherapy [19].

Human Ewing sarcoma cell line also formed spheres at a low frequency rate, which expressed higher levels of Oct3/4, Nanog, STAT3, Sox2, Sox10, and EWS-FLI1, and showed higher drug resistance, similarly to OS cells [19]. Similarly, chondrosarcoma cells that originated from patient biopsies formed spherical colonies which displayed Stro-1, CD44, and CD105, the mesenchymal stem cell markers [16]. However, Leuchte et al. demonstrated that sphere formation is not a reliable method to enrich CSCs of Ewing sarcoma, since these spheres did not continuously self-renew by secondary sphere formation, and sphere culture did not enhance the tumorigenicity in vivo [20]. Therefore, the sphere model might be inadequate to reflect CSC properties but at least useful for preclinical in vitro testing of novel therapies.

### 3.2. CD133 (Osteosarcoma, Ewing Sarcoma, and Chondrosarcoma)

CD133, first recognized as a novel antigen on CD34^+^ progenitor hematopoietic stem cells, is a glycoprotein with a five-transmembrane-domain protein encoded by the PROM1 gene [21–23]. Tirino et al. were the first to demonstrate that CD133^+^ osteosarcoma cells possessed CSC phenotypes. They showed that SaOS2, MG-63, and U2OS cell lines contained a small fraction of CD133^+^ cells ranging from 3% to 5%, which showed the following phenotypes: high proliferation rate, cell cycle detection in G_2_/M phase, positivity for Ki-67, formation of spheres, and inclusion of a small subset of SP cells (0.97%) [24]. They further identified that all human osteosarcoma and chondrosarcoma samples contained a small population of CD133^+^ cells [25]. Moreover, CD133^+^ cells of two stabilized cell lines from clinical samples (5.0%–7.8%) also showed self-renewability, sphere formation, adipogenic and osteogenic differentiation, high expression of stemness genes, and tumorigenicity [25].

Several molecules have been used in combination with CD133 expression as CSC markers. For example, Veselska et al. identified the cells expressing nestin in 18 osteosarcoma primary samples. Among 4 of the 18 stabilized cell lines, 3 contained nestin^+^/CD133^+^ cells at a frequency of 11%–100% [26]. In addition, He et al. added CD44 to their analysis and found that the CD133^+^CD44^+^ fraction was more aggressive regarding sphere formation, migration, and invasiveness than its counterparts and it showed the strongest potential for tumorigenicity and lung metastasis [27]. On the other hand, Ying et al. identified that CD49f^−^ fraction showed asymmetric division and contained a CD133^+^ fraction that increased in culture over time. Subsequently, CD49f^−^CD133^+^ fraction was revealed to show strong tumorigenicity correlated with an impaired osteogenic fate [28].

CD133^+^ fraction was also identified in Ewing sarcoma cells. Suvà et al. isolated CD133^+^ cells from three surgically resected tumors and revealed 4%–8% of bulk cell populations. These CD133^+^ cells displayed higher tumorigenicity, spherogenic potential, multipotency, and high expression levels of Oct-4 and Nanog [29]. On the other hand, Jiang et al. reported that CD133^+^ cells were identified in only 4 of 48 primary tumor specimens [30]. Among these four patients with CD133^+^, two were drug-resistant, but the others were event-free survivors. Moreover, no differences were observed in the drug resistance or tumorigenicity between CD133^+^ and CD133^−^ cells, except for the STA-ET-8.2 cell line [30]. Therefore, their study suggests that CD133 is significant as a CSC marker only in some cases of Ewing sarcoma.

### 3.3. Side Population Cells (Osteosarcoma, Ewing Sarcoma, and Chondrosarcoma)

A subpopulation that effluxes the DNA-binding dye Hoechst 33342 out of the cell membrane through an ATP-binding cassette (ABC) transporter was recognized as a stem cell population in analyses of hematopoietic stem cells [31, 32]. This cell population expressing the ABC transporter is defined as side population (SP) cells, which are distinguished from cells of the other population, called main population (MP) cells [33]. Wu et al. reported that SP cells were identified in five surgically excised osteosarcoma samples and two chondrosarcoma samples and showed higher tumorigenicity than MP cells [34]. SP cells were negative for two cases of grade 1 chondrosarcoma. SP cells were also identified at a frequency of 1.2% from Ewing cell line SK-ES-1 cells. SP cells regenerated both SP and non-SP cells and showed higher clonogenicity, drug resistance, and invasiveness than non-SP cells [35].

However, SP cells were detected in only one osteosarcoma and Ewing sarcoma cell line, respectively, and tumorigenicity of these cells was controversial [33, 36]. Therefore, further analyses including serial transplantation experiments are needed to determine the significance of SP cells as a bone sarcoma stem cell fraction [37]. Moreover, tumor cells resistant to Hoechst 33342 dye do not necessarily show tumorigenicity and metastatic ability [38], and cytometry gating used to isolate SP cells lacks the consistency of gating strategies used in marker staining [39], indicating the controversy of SP cells as a CSC fraction. Sun et al. confirmed that primary osteosarcoma specimens contained approximately 3.9% fraction of SP cells and identified the high expression of CD248 (endosialin), and other stem cell markers such as Oct3/4, Nanog, nestin, and CD133 [40]. SP/CD248^high^ cells showed strong tumorigenicity and invasiveness [40], indicating that CD248 is a potential therapeutic target.

### 3.4. CD117 and Stro-1 (Osteosarcoma)

CD117 and Stro-1 are expressed in mesenchymal stem cells [41]. CD117^+^Stro-1^+^ cells of osteosarcoma showed CSC phenotypes with high invasiveness and drug resistance. Adhikari et al. found that the CD117^+^Stro-1^+^ fraction of murine cell lines had differentiation ability, highly expressed CXCR4 and ABCG2, and showed higher drug resistance, tumorigenicity, and metastatic ability, than CD117^−^Stro-1^−^ cells [41]. These properties were validated in human osteosarcoma cell lines, suggesting that CD117^+^Stro-1^+^ cells possess CSC properties. However, the reliability of CD117^+^Stro-1^+^ in clinical samples remains controversial. Tirino et al. reported that all clinical samples in their analysis were negative for CD117, indicating that further investigation and discussion are necessary to confirm its potential as a CSC marker [25].

### 3.5. ALDH (Osteosarcoma and Ewing Sarcoma)

ALDH is a detoxifying enzyme responsible for the oxidation of intracellular aldehydes [42], which has been reported to play a role in the early differentiation of stem cells in the oxidation of retinol to retinoic acid [43]. High ALDH activity has been observed in murine and human hematopoietic and neural stem and progenitor cells [44, 45], and the evaluation of ALDH activity has been a useful approach in the isolation of CSCs in several tumors [45, 46]. Wang et al. reported that highly aggressive OS99-1 contained cells with high ALDH activity (ALDH^br^ cells) at a rate of 45.1%, while the corresponding rates were 1.8% in HuO9, 1.6% in SaOS2, and 0.6% in MG63 [47]. ALDH^br^ cells from a xenograft showed higher proliferation rate, clone formation, expression of Oct3/4A, Nanog, and Sox2, and tumorigenicity than ALDH^lo^ cells. Honoki et al. reported the similar results in spheres, which displayed stronger drug resistance than monolayer adherent cells [48]. Awad et al. demonstrated that Ewing sarcoma ALDH^high^ cells were enriched for clonogenicity, sphere formation, the expression of Oct3/4, Bmi-1, and Nanog, drug resistance, and tumorigenicity [46]. Notably, ALDH^high^ cells were resistant to doxorubicin but sensitive to YK-4-279, a small-molecule inhibitor of EWS-FLI1 [46]. YK-4-279 blocks oncogenic activity of EWS-FLI1 by blocking its interaction with RNA helicase A (RHA) and induces apoptosis in Ewing sarcoma cells both in vitro and in vivo. However, ALDH^high^ activity was not investigated in human specimens; therefore, future studies will be needed to evaluate the significance of ALDH^high^ cells and their clinicopathological correlations in Ewing sarcoma.

### 3.6. CBX3 and ABCA5 (Osteosarcoma)

Saini et al. confirmed that spheres from clinical specimens showed self-renewal, clonogenic, and tumorigenic potential and identified that they had lower expression levels of CD326, CD24, CD44, and higher ABCG2 [49]. In addition, proteomic and transcriptome analyses revealed high expression of chromobox protein homolog 3 (CBX3) and ABCA5, respectively, which was significantly higher in osteosarcoma than in primary osteoblast.

### 3.7. CD47 (Osteosarcoma)

CD47 is a transmembrane protein that acts as a self-signal on normal cells by inhibiting macrophage phagocytosis [50], and high expression of CD47 is a poor prognostic factor [51]. Xu et al. identified that CD47 protein was highly expressed in tumor tissue compared with that in normal controls and that the majority of CD44^+^ cells expressed CD47 (80%–99%) [52]. Interestingly, CD47 blockage increased the macrophage phagocytosis of tumor cells, indicating that this could be an effective immunotherapeutic approach.

### 3.8. CD271 (Osteosarcoma)

CD271 is one of the cell surface markers of bone marrow MSCs and was identified as a marker of melanoma CSCs [53]. Tian et al. found that CD271 was expressed in tumor tissues (0%–29%) and in a small population in osteosarcoma cell lines (5.4%–9.7%). CD271^+^ cells displayed the properties of self-renewal, differentiation, drug resistance, and tumorigenicity and showed higher expression of Oct3/4, Nanog, Stat3, Bcl-2, and ABCG2 than CD271^−^ cells [54].

### 3.9. CD57 (Ewing Sarcoma)

Wahl et al. investigated whether CD57 (HNK-1), a surface marker for migrating and proliferating neural crest cells, could be a CSC marker in Ewing sarcoma [55]. The CD57 expression level positively correlated with sphere formation. CD57^high^ cells were adhesive, invasive, and tumorigenic compared with CD57^low^ cells. However, the aggressiveness of CD57^high^ cells could not be solely attributed to the coexpression of CD133 since CD57^high^ VH-64 cells did not express CD133, while CD57^high^ WE-68 cells were also positive for CD133 [55].

## 4. Signaling Pathways Activated in Bone Sarcoma Stem Cells

Since most of the CSC markers summarized in the previous section are expressed on the normal cells and tissues, a comprehensive understanding of the tumor-specific molecular pathways underlying CSCs is needed for therapeutic application. Analyses of the signaling pathways of CSCs have been performed with the isolation technique based on the CSC markers in Table 1. Diverse molecular pathways such as self-renewal and epigenetic alterations have been documented, which are further investigated as therapeutic experiments (Figure 1, Table 2).

### 4.1. Self-Renewal Marker Genes (Oct3/4, Nanog, and Sox2)

The transcription factors Oct3/4, Sox2, and their target Nanog are known as key regulators of pluripotency [56]. Aberrant expression of Oct3/4 and Nanog has also been suggested to fulfill an oncogenic role in tumorigenesis and the development of CSCs [57, 58]. Levings et al. found that osteosarcoma cells derived from biopsy samples contained a small population of self-renewing spherical clones that showed significant increases of Oct-4 and Nanog expression. Tumorigenic OS521 cells were engineered to activate an Oct-4 promoter/GFP reporter and the GFP^+^ cells were at least 100-fold more tumorigenic and metastatic, capable of forming tumors at less than 300 cells, whereas only 1 of 8 mice developed a tumor at 3,000 cells in the GFP-depleted group [57, 58]. OS521Oct4-pGFP^+^ cells were capable of self-renewal in several passages, forming heterogeneous tumors for Oct-4/GFP expression. Basu-Roy et al. found that Sox2 mRNA and protein were highly expressed in human and murine osteosarcoma cell lines as well as osteosarcoma tissue samples at variably high levels [59]. Sox2 depletion by shRNA decreased colony formation in soft agar, migration, invasion, and tumorigenicity. High Sox2 expression was accompanied by reduced Wnt signaling, while the activation of Wnt signaling resulted in low Sox2 expression [59], suggesting that the activation of Wnt signaling antagonizes the effect of Sox2 in maintaining osteosarcoma cells.

### 4.2. MAPK, Wnt/*β*-Catenin, and Notch Pathway

The mitogen-activated protein kinase (MAPK) pathway is frequently activated in human cancers, including osteosarcoma [60]. Gemei et al. performed proteomic analysis using stem-like 3AB-OS cells and determined that the ERK/MAPK pathway is associated with tumorigenicity and self-renewability [61].

The Wnt signal transduction pathway coordinates myriad activities, from development and differentiation to proliferation and tumorigenesis [62]. Aberrant Wnt signaling has been reported in various tumors and shown to be associated with CSC activity [63]. Martins-Neves et al. reported that tumorigenic osteosarcoma spheres overexpressed SOX2 and KLF4 and showed specific activation of Wnt/*β*-catenin signaling [64]. In addition, Yi et al. confirmed that SP cells were contained in osteosarcoma samples and *β*-catenin and cyclin D1 were highly upregulated in them, indicating that this pathway is a potential target of novel anticancer drugs for osteosarcoma stem cells [65].

Notch signaling plays a key role in the normal development of many tissues and cell types through diverse effects on cell fate, stem cell renewal, differentiation, survival, and proliferation [66]. This signaling pathway functions as an oncogene or a tumor suppressor, depending on the cellular context [67], and is associated with osteosarcoma and Ewing sarcoma [68]. Mu et al. reported that ALDH activity in highly metastatic K7M2 cells is reduced by Notch inhibition and is also associated with increased resistance to oxidative stress, migration, invasion, and VEGF expression, suggesting that ALDH activity may be regulated by Notch signaling [69].

### 4.3. TGF-*β*, TNF-*α*, and BMP-2

Transforming growth factor-beta (TGF-*β*) is a pleiotropic cytokine that helps to maintain homeostasis, limits the growth of epithelial, endothelial, neuronal, and hematopoietic cell lineages, and acts as a mediator on tumors to promote further tumor expansion. Zhang et al. reported that TGF-*β*1 signaling and a hypoxic environment induced CSC phenotypes in a non-CSC population and that the blockage of TGF-*β*1 signaling inhibited the dedifferentiation and clonogenicity of osteosarcoma cells and reduced CSC self-renewal capacity, suggesting that CSCs may be yielded from differentiated cells [70].

Tumor necrosis factor-*α* (TNF*α*) is an inflammatory cytokine produced by macrophages/monocytes during acute inflammation and is responsible for a range of signaling events, leading to necrosis or apoptosis. In addition, Mori et al. reported that TNF*α* is required for the tumorigenesis of osteosarcoma. Lethal tumorigenesis of AX osteosarcoma cells was completely abrogated in TNF*α*-deficient mice and IL-1a/IL-1b doubly deficient mice, which occurred through ERK activation [71].

Bone morphogenetic proteins (BMPs) are members of the TGF-*β* superfamily, which play pivotal roles in not only bone and cartilage formation but also cell proliferation, apoptosis, differentiation, and tumorigenesis. Wang et al. evaluated the effect of BMP2 on ALDH^br^ OS cells and determined that it suppresses tumor growth by reducing the expression of Oct3/4, Nanog, and Sox-2 genes and inducing the differentiation markers Runx-2 and collagen type I [72].

### 4.4. Epigenetic Regulators in CSCs

#### 4.4.1. MicroRNA (miR-29b, 133a, 143, 145, and 1247)

miRNAs are small regulatory RNA molecules that modulate the expression of their target genes and play important roles in a variety of physiological processes, such as development, differentiation, cell proliferation, apoptosis, and stress responses [73]. In recent years, many miRNAs have been investigated in various malignant diseases [74]. Deregulation of the expression of miRNAs has been shown to contribute to cancer development through various mechanisms, including deletions, amplifications, or mutations involving miRNA loci, epigenetic silencing, and the dysregulation of transcription factors that target specific miRNAs [75]. Recently, miRNAs have been focused on as a novel approach for regulating the phenotypes of CSCs of bone sarcomas. Di Fiore et al. identified that miR-29b-1 suppressed the stemness properties of 3AB-OS CSCs, including sphere/colony formation and drug resistance, but did not their invasive and migratory abilities [76]. Our microarray analysis revealed upregulated miR-133a expression levels in the CD133^high^ fraction. High expression levels of miR-133a and low expression levels of its target genes were significantly correlated with clinical prognosis, and the silencing of miR-133a contributed to cell invasion and lung metastasis [77]. Zhou et al. found that tumor-suppressive miR-143 was downregulated in CD133^+^ALDH^+^ cells and associated with drug resistance and autophagy. From a microarray analysis with CD117^+^Stro-1^+^ and CD117^−^Stro-1^−^ cells [78], Zhao et al. found significant downregulation of miR-1247, which targets MAP3K9, promoting OS proliferation and sphere formation [79].

Riggi et al. identified Ewing sarcoma stem cells using human pediatric MSCs (hpMSCs). They demonstrated that hpMSCs expressing EWS-FLI-1 (hpMSC^EWS-FLI-1^) generate a CD133^+^ subpopulation displaying CSC phenotypes [80]. Interestingly, EWS-FLI-1 induced the expression of Sox2, Oct-4, and Nanog through miR-145 repression, and they function in a feedback loop with their common target gene, Sox2, which regulates the differentiation and tumorigenicity of Ewing sarcoma cells. In addition, De Vito et al. added the molecular mechanisms underlying Ewing sarcoma stem cells. They found that TARBP2 (TAR RNA-binding protein 2), which forms part of the Dicer-1 complex, was suppressed in CD133^+^ Ewing sarcoma cells as well as hpMSC^EWS-FLI-1^, and enoxacin, which augments TARBP2 function, inhibited tumor growth through the restoration of miRNA maturation [81]. Similarly, the systemic injection of 30 *μ*g of synthetic TARBP2-dependent miR-143 or miR-145 showed a significant reduction of tumor volume, suggesting that CSC phenotypes of Ewing sarcoma correlate with the deregulation of TARBP2-dependent miRNA expression [81].

#### 4.4.2. Long Noncoding RNA (HIF2PUT)

Among the various types of noncoding RNA, miRNAs have been most extensively studied and their role in carcinogenesis has been established, but several long noncoding RNAs (lncRNAs) with functional involvement in malignant diseases have also been identified. Wang et al. investigated the function of hypoxia-inducible factor-2*α* (HIF-2*α*) promoter upstream transcript (HIF2PUT) in osteosarcoma stem cells. The proportion of CD133^+^ cells decreased and inhibition of sphere formation, proliferation, and migration was decreased by the overexpression of HIF2PUT, while the knockdown of HIF2PUT showed the opposite function via alteration of HIF-2*α* mRNA expression [82].

#### 4.4.3. Telomerase

Human telomerase is a reverse transcriptase composed of a catalytic component, telomerase reverse transcriptase (TERT), and a telomerase RNA component (TERC) [83]. In most normal human somatic cells, telomerase activity is undetectable, while stem/progenitor cells express telomerase, and its activity is also detected in most malignant cancers [84]. Yu et al. identified high telomerase activity in sphere-derived osteosarcoma cells (TEL^+^) and TEL^+^ cells showed increased sphere and tumor-propagating capacity, invasiveness, metastatic activity, and drug resistance. Treatment with MST312, a telomerase inhibitor, was also shown to target TEL^+^ cells and prevent the tumorigenicity of osteosarcoma cells [85].

#### 4.4.4. TSSC3

Huang et al. found that overexpression of the imprinted gene TSSC3 (tumor-suppressing STF cDNA 3), an apoptosis-related gene, was associated with growth inhibition and apoptotic induction in osteosarcoma; they then further analyzed the effect of this on osteosarcoma stem cells [86]. The expression of TSSC3 was low in osteosarcoma spheres and the overexpression of TSSC3 downregulated the expression of the stem cell markers Oct3/4, Nanog, and Sox2, decreased the clone formation rate, and induced apoptosis, suggesting that TSSC3 plays a suppressive role in osteosarcoma spheres [86].

## 5. Therapeutic Targeting of Bone Sarcoma Stem Cells

Preclinical studies of the therapeutic application to target bone sarcoma CSCs have now been performed on the basis of studies concerning the molecular biology underlying CSCs. Strategies include using the molecular target drug inhibiting NF-*κ*B, PI3K, HDAC, or a “next generation” nucleic-acid therapeutics (Figure 1, Table 3). The information on these compounds is partly available from the https://ClinicalTrials.gov/ website. Although there are little compounds that have been approved for clinical use, several candidates have already been tested in promising trials.

### 5.1. Inhibitor of PI3K Signaling Pathway

The phosphatidylinositol 3-kinase (PI3K) signaling pathway has inherent oncogenic potential and is involved in CSC biology in several malignancies including breast, colon, pancreas, brain, and bladder [87]. Gong et al. investigated the efficacy of LY2940002, a PIK3 inhibitor that prevents the phosphorylation of protein kinase B, and revealed that this compound increased the number of cells in the G_0_/G_1_ phase and induced apoptosis via the activation of caspase-3, caspase-9, and PARP in osteosarcoma stem cells [88]. Preclinical experiments with in vivo models have not been completed, although a phase I study of SF1126, a novel inhibitor of PI3 kinase and mTOR that includes an active moiety consisting of LY294002, for patients with relapsed or refractory neuroblastoma is reportedly ongoing [89].

### 5.2. Inhibitor of NF-*κ*B Signaling Pathway

Nuclear factor *κ*B (NF-*κ*B) comprises a family of transcription factors involved in the regulation of various biological responses. NF-*κ*B plays an important role in the regulation of immune responses and inflammation but accumulated evidence has shown that it also plays a major role in oncogenesis. There is evidence that NF-*κ*B-regulated gene products play a major role in inhibiting apoptosis in leukemic stem cells [90]. Mongre et al. investigated the efficacy of BRM270, a well-known traditional Chinese medicine, against stem-like cancer-initiating cells; this compound selectively inhibited NF-*κ*B transcriptional activity, resulting in decreased expression of interleukin-6, which is a cytokine associated with metastasis. BRM270 induced IL-6-mediated apoptosis in CD133^+^ osteosarcoma stem cells via the downregulation of chromatin SMC2 [91]. However, at present, there is no information on its clinical use or trials in CSC therapy [92].

### 5.3. HDAC Inhibitor

Epigenetics is defined as heritable changes in gene expression that are not accompanied by changes in the DNA sequence; it includes DNA methylation, histone modifications, nucleosome positioning, and noncoding RNAs. Histone deacetylase (HDAC) inhibitors have been reported to suppress CSC phenotypes in solid malignancies [93, 94]. Di Pompo et al. tested HDAC inhibitors on sarcospheres of osteosarcoma, Ewing sarcoma, and rhabdomyosarcoma and reported that MC1742 and MC2625 inhibited sphere growth by inducing apoptosis with increased acetyl-H3 and acetyl-tubulin levels [95]. However, in vivo preclinical sarcoma models have not been yet established. Although there is no information on clinical trials for these compounds, several epigenetic drugs including azacitidine, decitabine, vorinostat, and romidepsin have already received FDA approval and undergone clinical trials for osteosarcoma, Ewing sarcoma, and other soft-tissue sarcomas [92].

### 5.4. MicroRNA Therapeutics

miRNAs provide new therapeutic targets for many diseases and recent progress in the development of effective strategies to adjust miRNA dysregulation has indicated their potential for clinical application [96]. Synthetic molecular mimics of tumor suppressor miRNAs or the inhibition of oncogenic miRNAs by chemically modified antisense oligonucleotides (ASOs) have been widely tested in preclinical trials. Chemical modifications, including 2′-O-methyl, 2′-O-methoxyethyl, 2′-fluoro, and locked nucleic acid (LNA), have improved the stability, biodistribution, and delivery of ASOs. We have performed preclinical evaluation of systemic LNA treatment for osteosarcoma stem cells. Among three upregulated miRNAs in the SaOS2 CD133^high^ population, miR-133a regulated cell invasion and the upregulated levels were significantly correlated with poor prognosis of patients [77]. Inversely, the silencing of miR-133a with LNA reduced cell invasion and the systemic administration of LNA along with cisplatin suppressed lung metastasis. The tumor expression levels of miR-133a were reduced by LNA administration without a drug delivery system. Currently, a multicenter phase I study involving the liposomal injection of MRX34, an miR-34 mimic, for 5 days with 2 weeks off, is in progress for patients aged over 18 years with unresectable primary liver cancer, advanced metastatic cancer with or without liver metastasis (melanoma, lung cancer), lymphoma, and multiple myeloma. LNA has also undergone a clinical phase II trial as miravirsen (SPC3649) for chronic hepatitis C (HCV). Miravirsen works mainly by hybridizing with mature miR-122, a liver-specific miRNA with an important role in the life cycle of HCV, and also binds to the stem-loop structure of pri- and pre-miR-122 [97]. To date, this compound has not been tested for malignant diseases [92].

### 5.5. Salinomycin

Salinomycin is a polyether ionophore antibiotic, which has been widely used as an anticoccidiosis agent in chickens [87]. It was identified in a chemical screen that had been designed to discover compounds with selective toxicity for breast CSCs [98]. Tang et al. identified that salinomycin inhibited osteosarcoma by selectively targeting CSCs both in vitro and in vivo without severe side effects [99]. They further identified that downregulation of the Wnt/*β*-catenin self-renewal pathway might contribute to the inhibitory effects of salinomycin on osteosarcoma stem cells [99]. However, salinomycin displays poor aqueous solubility that hinders its clinical application [89]. Thus, Ni et al. developed salinomycin-loaded PEGylated polynanoparticles conjugated with CD133 aptamers (Ap-SAL-NP) and evaluated their cytotoxicity to CD133^+^ cells. Ap-SAL-NP exhibited specific cytotoxicity toward SaOS2 CD133^+^ cells and intravenous injections via the tail vein of tumor-bearing mice exhibited significant antitumor activity compared with salinomycin and a control compound [89]. However, there does not seem to be any information on clinical trials of these compounds.

### 5.6. Bufalin

Bufalin is the active ingredient of the Chinese medicine Chan Su, which is extracted from dried toad venom from the skin glands of* Bufo gargarizans* [100]. This compound inhibits the proliferation of hepatocellular carcinoma and induces apoptosis in cancer cell lines of leukemia, prostate cancer, gastric cancer, and osteosarcoma [100]. Chang et al. identified that bufalin induced the shrinkage of tumor spheres via the activation of caspase-3 and downregulated stem cell markers including ALDH1, TERT, Nanog, CD133, Notch, and Bmi1. miR-148 was found to be a target of bufalin, which regulates DNMT1 and p27 [100]. A clinical trial of huachansu, which includes bufalin with gemcitabine, for pancreatic cancer has been completed, but the results are not currently available [92].

## 6. Conclusions and Perspectives

Substantial effort has been expended on the identification and characterization of bone sarcoma stem cells. To date, various CSC markers have been identified especially in osteosarcoma, which might reflect the histological and genetic heterogeneity of this tumor. Thus, a definitive marker for osteosarcoma stem cells remains to be found and there seems to be heterogeneity even within the isolated CSC fraction. Furthermore, only conventional CSC markers have been analyzed for chondrosarcoma. Therefore, we have to conclude that CSC research in bone sarcomas is still at an immature stage, and further research is required to fully understand the CSC markers and their functions. In addition, no investigations have been performed on the correlation between CSC markers and the drug-resistant fractions within clinically resected specimens after neoadjuvant chemotherapy or metastatic specimens at the early phase of tumor formation. Further investigation using a number of clinically resected specimens and elucidation of the clinical relevance will be important for the precise characterization of bone sarcoma stem cells.

To date, most reports have focused on the common CSC markers such as CD133 or ALDH, which would be reasonable if the sarcoma stem cell subpopulations emerge after the accumulation of further epigenetic or genetic alterations in a subset of tumor cells. However, if sarcomas are derived from a single cell that is transformed into a sarcoma stem cell, the mesenchymal stem cell markers such as CD117/Stro-1 or CD271, and a neural crest cell marker such as CD57, would be reasonable for sarcomagenesis. Nevertheless, the positivity rate of these mesenchymal stem cell markers in the clinical specimens was negative to quite low [25], indicating that further investigation is necessary to confirm their potential as bone stem cell markers. Despite the inconsistency in the markers of bone sarcoma stem cells, clinical problems that must be overcome are drug resistance and metastasis. The molecular regulations that modulate these phenotypes with clinicopathological relevance, such as Wnt/*β*-catenin and miRNAs, might be important. Some of the tumor-suppressive miRNAs directly regulate the oncogenic fusion gene EWS-FLI-1 and indirectly target CSC markers (Figure 1), thereby anticipating their clinical application as miRNA therapeutics. Clinical benefit will be provided by anti-CSC compounds that possess a broad spectrum to the lethal phenotypes and are of prognostic significance.

Among the anti-CSC compounds tested in the reported studies, some have already undergone clinical trials for other malignant diseases, which will also be promising for sarcoma patients. The anti-CSC compounds under clinical trials for other malignancies but not for bone sarcomas include ROR1 inhibitor, oncolytic adenovirus, and immunotherapy [87]. Receptor tyrosine kinase-like orphan receptor 1 (ROR1), a tyrosine kinase-like cell surface protein that is expressed during embryogenesis, is associated with Ewing sarcoma as well as breast and ovarian cancer CSCs [101, 102]. Cirmtuzumab, which binds with high affinity to ROR1, is currently being investigated in patients with chronic lymphocytic leukemia who are ineligible for chemotherapy [87, 102]. A telomerase-specific oncolytic adenovirus (OBP-301) has already been reported to be effective for the treatment of bone and soft-tissue sarcomas [103]. Indeed, this oncolytic virus was previously confirmed to be effective by targeting gastric CSCs [104]. Kano et al. found that the SP cells showed the expression of human leukocyte antigen (HLA) class I molecules on the cell surface, and the CTL clone Tc4C-6, induced by mixed lymphocyte tumor cell culture using autologous peripheral blood mononuclear cells and freshly isolated SP cells, showed specific cytotoxicity against the SP cells [105]. These compounds could be candidates to overcome bone sarcoma stem cells but have not yet been tested preclinically for bone sarcomas.

Current clinical trials will clarify the efficacy of the anti-CSC compounds in the near future. Since these compounds are effective at least preclinically, their drug screening in vivo should clarify which are the most effective and the least toxic. However, a difficult issue is how to confirm that these anti-CSC compounds are clinically effective for CSCs. Indeed, these compounds have been clinically applied in combination with current chemotherapeutic drugs. If a favorable outcome were observed, the trial would be a clinical success. Then, researchers would have to carefully investigate the effect on CSCs using clinical specimens to confirm the efficacy of anti-CSC compounds. At this stage, the inconsistency of CSC markers is a major problem, and the development of a novel biomarker for detecting CSCs is required. Liquid biopsy technologies including circulating tumor cells, circulating cell-free DNA, circulating cell-free miRNA, and circulating exosomes might solve these problems as noninvasive biomarkers [106], which have not yet been analyzed using clinical samples from patients with bone sarcomas.

Since bone sarcoma is a rare malignant disease, global cooperation among basic researchers and clinicians will be required to improve its prognosis. Although CSC studies on bone sarcoma are in their infancy compared with studies on other malignancies, the development of anti-CSC compounds is highly anticipated. These trials should yield novel treatment strategies for bone sarcoma patients against a background in which the clinical outcomes have almost plateaued for 10 years.

## Figures and Tables

**Table 1:** Markers of bone sarcoma stem cells.

Subtype	Marker	Function, clinical relevance	Reference
Osteosarcoma	Sarcosphere	Drug resistance, overexpressing Oct3/4, Nanog, and Stat3	[16–19]
	CD133	Sphere formation, multipotency, tumorigenicity, self-renewal, inclusion of SP cells, overexpressing Oct3/4 and Nanog	[24–26]
	/CD49f^−^	Sphere formation, migration, invasion, tumorigenicity, lung metastasis	[28]
	/CD44	Sphere formation, migration, invasion, tumorigenicity, lung metastasis	[27]
	CD117/Stro-1	Drug resistance, invasion, metastasis, tumorigenicity, self-renewal, overexpressing ABCG2 and CXCR4	[25, 41]
	SP	Drug resistance, self-renewal, tumorigenicity	[34, 35]
	/CD248	Tumorigenicity, invasion	[40]
	ALDH	Proliferation, tumorigenicity, overexpressing Oct3/4, Nanog, Sox2, and Stat3	[47, 48]
	CBX3 and ABCA5	Highly expressed in spheres	[49]
	CD47	Invasion, blockage of macrophage phagocytosis, prognostic value	[52]
	CD271	Self-renewal, differentiation, drug resistance, tumorigenicity, overexpressing Oct3/4, Nanog, Stat3, Bcl-2, and ABCG2	[54]
	Oct3/4	Tumorigenicity, self-renewal	[57, 58]
	Sox2	Soft agar growth, migration, invasion, tumorigenicity, reduced Wnt signaling	[59]

Ewing sarcoma	Sarcosphere	Drug resistance, overexpressing Oc3/4, Nanog, Stat3, Sox2, Sox 10, and EWS-FLI1, fail to self-renew and enhance tumorigenicity	[19, 20]
	CD133	Sphere formation, multipotency, tumorigenicity, overexpressing Oct3/4 and Nanog, no difference in drug resistance and tumorigenicity	[29, 30]
	CD57	Migration, invasion, multipotency, tumorigenicity, no correlation with CD133	[55]
	SP	Drug resistance, clonogenicity, invasion, asymmetric division	[33, 36, 37]
	ALDH	Clonogenicity, sphere formation, tumorigenicity, drug resistance	[46]

Chondrosarcoma	Sarcosphere	Multipotency, overexpressing Oct3/4, Nanog, and Stat3, expressing Stro-1, CD44, and CD105	[16]
	CD133	Tumorigenicity	[25]
	SP	NA	[34]

SP: side population; ALDH: aldehyde dehydrogenase; NA: not available.

**Table 2:** Regulators of bone sarcoma stem cells.

Subtype	Category	Regulators	Target CSC	Function, clinical relevance	Reference
Osteosarcoma	Signaling pathways	MAPK	3AB-OS	Self-renewal, tumorigenicity	[60, 61]
		Wnt/*β*-catenin	Sarcosphere, SP	Tumorigenicity, drug resistance, invasion	[64, 65]
		Notch	ALDH	Oxidative stress, migration, invasion, VEGF expression	[69]
		TGF-*β*	Sarcosphere	Dedifferentiation, clonogenicity	[70]
		TNF-*α*	AX	Tumorigenesis, differentiation	[71]
		BMP2	ALDH	Tumorigenesis, stem-cell marker, differentiation marker	[72]

Osteosarcoma	Epigenetic regulators	miR-29b	3AB-OS	Sphere, clonogenicity, drug resistance	[76]
		miR-133a	CD133	Invasion, metastasis, prognosis	[77]
		miR-143	CD133/ALDH	Drug resistance, autophagy, prognosis	[78]
		miR-1247	CD117/Stro-1	Sphere, proliferation	[79]
		HIF2PUT	CD133	Sphere, proliferation, migration, alteration of HIF2-*α* mRNA	[82]
		Telomerase	Sarcosphere	Sphere, invasion, drug resistance, tumorigenicity	[85]
		TSSC3	Sarcosphere	Sphere, clonogenicity, apoptosis, regulate Oct3/4, Nanog, and Sox2	[86]
		MLH1, MSH2	Sarcosphere	Drug resistance	[19]

Ewing sarcoma	Epigenetic regulators	TARBP2	CD133	Tumorigenicity, CD133^+^ frequency	[81]
		miR-145	CD133	Tumorigenicity	[80]
		miR-143, -145	CD133	Clonogenicity, tumorigenicity	[81]

**Table 3:** Preclinical trials of novel agents targeting bone sarcoma stem cells.

Subtype	Agents	Function	Target CSC	Mechanism	Reference
Osteosarcoma	LY294002	PIK3 inhibitor	Sarcosphere	Increase the number of cells in G_0_/G_1_ phase and induction of apoptosis via activation of caspase-3, caspase-9, and PARP	[87]
	BRM270	NF-*κ*B inhibitor	CD133	Induce IL-6 mediated apoptosis in osteosarcoma CD133^+^ cells via downregulation of chromatin SMC2	[91]
	MC1742	HDAC inhibitor	Sarcosphere	Inhibit sphere growth of osteosarcoma and Ewing sarcoma by apoptosis induction with increased acetyl-H3 and acetyl-tubulin levels	[95]
	LNA-133a	miR-133a inhibitor	CD133	Inhibit invasion and metastasis of osteosarcoma CD133^+^ cells via several target genes including ANXA2	[77]
	Salinomycin	Antibacterial agent	Sarcosphere	Impair Wnt/*β*-catenin signaling by degradation of *β*-catenin	[99]
	Ap-SAL-NP	Nanoparticle	CD133	Selectively kill CD133^+^ cells by salinomycin-loaded PEGylated polynanoparticles conjugated with CD133 aptamer	[89]
	Bufalin	Unknown	Sarcosphere	Induce shrinkage of tumor spheres via activation of caspase-3 and downregulate stem cell markers, targeting miR-148	[100]

Ewing sarcoma	YK-4-209	EWS-FLI1 inhibitor	ALDH	Block RNA helicase A (RHA) binding to EWS-FLI1	[46]
	Enoxacin	Antibacterial agent	CD133	Augment TARBP2 expression, which is repressed in CD133^+^ Ewing cells, and reduce CD133^+^ subpopulation through restoration of miRNA expression	[81]
	miR-143,145	Synthetic miRNA	CD133	Repress the expression of target genes Oct3/4, Sox2, as well as EWS-FLI1	[81]
